# Do laypersons perceive aesthetic differences between coated and uncoated orthodontic archwires?

**DOI:** 10.1590/2177-6709.24.1.062-067.oar

**Published:** 2019

**Authors:** Célia Regina Maio Pinzan-Vercelino, Ricardo Gabriel Calvet Campelo, Claudia Gonçalves Fahd, Meire Coelho Ferreira, Melissa Proença Nogueira Fialho, Júlio de Araújo Gurgel

**Affiliations:** 1 Universidade Ceuma, Departamento de Odontologia (São Luís/MA, Brazil).; 2 Universidade Estadual Paulista, Departamento de Fonoaudiologia (Marília/SP, Brazil).

**Keywords:** Esthetics, Orthodontic wires, Visual perception, Orthodontics, corrective

## Abstract

**Introduction:**

Aesthetic brackets are routinely combined with metallic wires in fixed orthodontic therapy, mainly due to the disadvantages of the clinical use of aesthetic archwires. The current situation needs to be explored in the literature by considering laypersons’ perceptions.

**Objective:**

The objective of this cross-sectional study was to evaluate laypersons’ aesthetic perceptions of metal archwires with and without aesthetic coating. Three age ranges and both sexes were evaluated.

**Methods:**

A volunteer using fixed aesthetic orthodontic appliance was photographed wearing the following archwires: stainless steel, nickel-titanium (NiTi), NiTi coated with epoxy resin and NiTi coated with rhodium. Using a 100-mm visual analog scale, 90 laypersons evaluated the photographs. Sex and age ranges (18-30, 31-45, over 46 years of age) were evaluated. The comparisons between the archwires and between age ranges were made using ANOVA and Tukey’s test. Genders were compared using Mann-Whitney test.

**Results:**

The results showed that the evaluators considered the archwire coated with epoxy resin to be the most aesthetic (60.64 ± 13.04) and the NiTi wire to be the least aesthetic (30.82 ± 7.79) (*p*< 0.05). Only the range of 31-45 years of age considered the NiTi archwires less aesthetic, when compared with the other age groups. For the other archwires, no statistically significant difference were found between the age groups. No differences between the sexes were detected.

**Conclusions:**

The results indicated that the aesthetic coated archwires represent an improvement in the visual aspect of ceramic brackets. The epoxy-coated metal wire was considered the most aesthetic option.

## RESUMO

**Introdução:**

rotineiramente na terapia ortodôntica fixa, utilizam-se braquetes estéticos combinados com fios metálicos, principalmente devido a algumas desvantagens no uso clínico dos fios estéticos. A percepção estética dos leigos nessa situação precisa ser explorada na literatura.

**Objetivo:**

o objetivo deste estudo transversal foi avaliar a percepção estética dos leigos em relação aos fios ortodônticos metálicos recobertos ou não por materiais estéticos. Três faixas etárias, dos dois sexos, foram avaliadas.

**Métodos:**

uma paciente usando aparelho ortodôntico fixo estético foi fotografada com os seguintes fios ortodônticos: aço inoxidável, níquel-titânio (NiTi), NiTi recoberto por resina epóxica e NiTi recoberto por ródio. As fotografias foram avaliadas por 90 leigos, utilizando-se uma escala visual analógica de 100-mm. Ambos os sexos e diferentes faixas etárias (18-30, 31-45 e acima de 46 anos) foram avaliados. As comparações entre os fios e entre as faixas etárias foram realizadas aplicando-se os testes ANOVA e Tukey. Os sexos foram comparados utilizando-se o teste de Mann-Whitney.

**Resultados:**

os resultados demonstraram que os avaliadores consideraram o fio recoberto por resina epóxica como o mais estético (60,64 ± 13,04) e o fio de NiTi foi classificado como o menos estético (30,82 ± 7,79) (*p*< 0,05). Apenas a faixa etária de 31-45 anos considerou o fio de NiTi menos estético, quando comparada às outras faixas etárias; para os outros fios, não foi observada diferença estatisticamente significativa entre as faixas etárias. Não foi detectada diferença entre os sexos.

**Conclusões:**

os resultados indicaram que os fios estéticos recobertos melhoraram o aspecto visual dos braquetes cerâmicos. O fio metálico recoberto por resina epóxica foi considerado a opção mais estética.

## INTRODUCTION

Adult patients have restrictions on the use of fixed appliances and are more demanding in the aesthetic judgment regarding the use of these.^1^ ^-^ ^3^ Recent research has shown that individuals who self-assessed after bonding brackets judged themselves less attractive, particularly when metal brackets were used.^3^

Today, the available esthetic appliances comprise lingual appliances, plastic aligners and esthetic brackets, in both conventional and self-ligating systems. Even when the patient opts for the use of aesthetic brackets, orthodontic therapy is routinely performed with metallic wires, mainly due to problems related to aesthetic archwires’ color changes^4^ and coating instability.^5^ ^-^ ^6^ However, in some situations, patients desire enhanced aesthetics, particularly for social events such as weddings, graduation ceremonies, parties and employment interviews.

The appearance of the archwire is known to be irrelevant when a metal appliance is used.^2^ However, there is a question regarding whether there is a difference between coated and uncoated archwires in laypersons’ aesthetic perceptions. Aesthetic archwires are more expensive than uncoated archwires and have limited clinical performance; therefore, is it worth using them? Some researchers have evaluated aesthetic judgments related to various types of appliances available on the market;^3^ ^,^ ^7^ however, up to now, little attention has been paid to the aesthetic value of coated orthodontic archwires. To date, no studies have been published in the literature focusing the esthetics of orthodontic archwires. Therefore, the aim of this study was to evaluate laypersons’ aesthetic perceptions in relation to metal archwires with and without aesthetic coatings. Additionally, the aesthetic perceptions of archwires was compared between three age groups and between both sexes. 

## MATERIAL AND METHODS

This study had a cross-sectional design, and it was approved by the institutional review board of Centro Universitário do Maranhão/UNICEUMA (protocol #1.066.942). Written informed consent was obtained from all participants.

A pilot study was conducted to determine the appropriate sample size to compare the mean scores using the visual analog scale. The parameters used were a confidence level of 99%, power of 90%, standard deviation of 13.51, and a minimum difference of 10-mm in the mean scores between the groups (www.sealedenvelope.com/power/continuous-equivalence). The minimum number of evaluators was determined to be 68, and 20% was added to this value, thus resulting in the minimum number of 82 participants. 

The group of evaluators was heterogeneous and was represented by 90 layperson, who were unrelated to dentistry, and not linked to artistic activities. The evaluators were equally distributed in relation to sex (45 men and 45 women) and ages (30 individuals between 18 and 30 years old, 30 individuals between 31 and 45, and 30 individuals over the age of 46).^8^ ^-^ ^9^ The mean age of the evaluators was 37.8 years (standard deviation: 10.48; minimum: 23; and maximum: 57).

A volunteer using an aesthetic orthodontic appliance (ICeram, Orthometric, Ma’anshan, China) was photographed from a front view, at a standardized distance of one meter from the camera; in each photo, the volunteer’s head was at a 90^o^ angle in relation to the ground. The volunteer’s anterior teeth were healthy, had no restorations, and had an adequate height/width proportion in the aesthetic zone; the volunteer had a consonant smile arch and a gingival height of less than 1-mm. 

**Figure 1:**
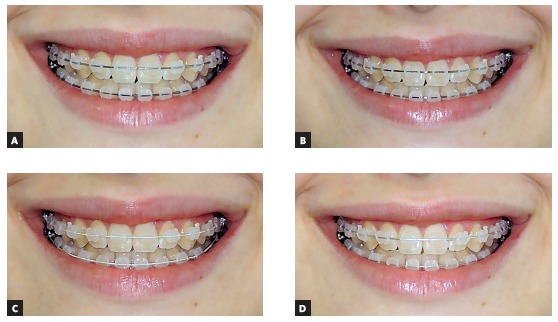
A) Stainless steel archwire. B) NiTi archwire. C) NiTi archwire coated with epoxy resin. D) NiTi archwire coated with rhodium.

Four photographs of the volunteer were taken, varying the installed orthodontic wire; all the archwires were 0.019 × 0.025-in and from the same commercial brand (Dentsply GAC International Inc., Bohemia, NY, USA). The archwires tested were: stainless steel, nickel-titanium (NiTi) (Neo-Sentalloy), NiTi coated with epoxy resin (Spectra Coated NiTi), and NiTi coated with rhodium (High Aesthetic Neo-Sentalloy) (Fig 1). The archwires were selected from among those available on the market at the beginning of the study. All the archwires were tied using aesthetic ligatures (Easy-To-Tie Obscure, 3M Unitek, Monrovia, CA, USA). The volunteer remained seated and was positioned in a cephalostat while the photographs were taken and during the clinical procedure of archwires change. 

The images were manipulated on a computer with the use of Adobe Photoshop CS4 (Adobe Systems, San Jose, Calif, USA). The images were retouched to adjust color, brightness and contrast. They were also condensed to achieve an image with measurements similar to those on the actual patient (the patient’s maxillary right central incisor was used as a reference).^2^ ^,^ ^10^ Most part of the nose, chin and cheeks were removed from the images to reduce factors that could have influenced the process of evaluating the images.^11^ The final images were presented in a standardized color and format, and with a resolution of 300 dpi. 

The images were printed and mounted in an album in random order. This album was presented to the evaluators, who were always in the presence of the same researcher. This researcher controlled the time of observation (30 seconds) for each image. Comparison among the photographs was not permitted.^12^

**Figure 2:**
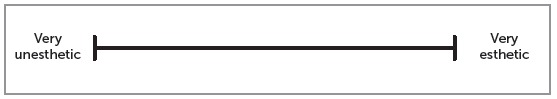
Visual analog scale (VAS). The evaluators were instructed to mark a point along the scale to indicate their aesthetic perceptions of each smile.

Each evaluator was given a brief explanation of the study and was asked to evaluate the appliance’ aesthetics of the images using the visual analog scale (VAS), which was printed separately for each of the images, as in previous studies.^7^ ^,^ ^10^ ^,^ ^12^ ^,^ ^13^ The visual analog scale consisted of a 100-mm uninterrupted line labeled “very unaesthetic” on the left side and “very aesthetic” on the right (Fig 2). The evaluators were instructed to make a vertical mark along the scale to indicate their aesthetic perceptions of each smile. The intensity of the evaluator’s assessment was measured to the nearest millimeter from the left extreme side to where the rater’s mark was made. The scores were measured in millimeters using an electronic digital caliper (Mitutoyo Digimatic Caliper 200 mm/.0005). 

### Statistical analysis

To evaluate intrarater agreement, two identical images were printed and mounted in the album together with the other photographs. The evaluators’ scores for these duplicate photographs were analyzed using the Intraclass Correlation Coefficient (ICC). 

To analyze the measurement reproducibility, the scales of 30 evaluators were re-measured after an interval of four weeks. A paired *t* test was applied with the purpose of evaluating the significance of the differences between the two measurements, thus demonstrating the systematic error. The Dahlberg formula (Se^2^= ∑d^2^/2n) was used to evaluate the casual error.

Descriptive statistics were reported as means and standard deviations. The measurements were submitted to the Kolmogorov-Smirnov test to evaluate the data distribution. To evaluate laypersons’ aesthetic perceptions of metal archwires with and without aesthetic coating, the one-way analysis of variance (ANOVA) with the Tukey *post-hoc* test were applied. These tests were also used to compare the three age groups. To evaluate the archwires’ aesthetic perception between genders, the Mann-Whitney test was applied. The level of significance was established at 5%. All analyses were performed with the SPSS statistical software program (version 21.0, IBM Corporation, Armonk, New York, USA).

## RESULTS

The evaluators’ aesthetic perceptions using the VAS scale showed satisfactory reproducibility (ICC = 0.682).^2^ No systematic error was detected (*t* test: p = 0.10), and the causal error was acceptable (Dahlberg = 0.207).

The descriptive statistics and the results of the ANOVA and Tukey’s test are demonstrated in Table 1. The results showed that the aesthetic archwires were rated higher, representing an improvement in the visual aspect of ceramic brackets. The epoxy-coated archwire produced the best aesthetic effect when analyzed by laypersons. The uncoated NiTi archwire was considered the least aesthetic.

**Table 1:** Descriptive statistics (mean and standard deviation) and comparisons (ANOVA and Tukey’s test) regarding laypersons’ perceptions of various orthodontic archwires.

	Steel archwire (Mean ± S.D.)	NiTi archwire (Mean ± S.D.)	Epoxy archwire (Mean ± S.D.)	Rhodium archwire (Mean ± S.D.)	p
Laypersons	36.83 ± 9.31^B^	30.82 ± 7.79^A^	60.64 ± 13.04^D^	43.40 ± 9.36^C^	<0.001

Different superscript capital letters represent statistically significant difference (Tukey test).

The laypersons’ aesthetic perceptions of metal archwires with and without aesthetic coating were similar in the age groups of 18-30 years and over 46 years (Table 2). Only the age group between 31 and 45 years considered the NiTi archwires less aesthetic when compared with the other age groups (Table 2). 

**Table 2:** Descriptive statistics (mean and standard deviation) and comparison between ages (ANOVA and Tukey’s test).

	18 - 30 years old (Mean ± S.D.)	31 - 45 years old (Mean ± S.D.)	> 46 years old (Mean ± S.D.)
Steel archwire	33.34 ± 8.24^A^	36.69 ± 9.06^A^	40.45 ± 9.39^A^
NiTi archwire	32.00 ± 6.15^A^	29.25 ± 8.33^B^	31.21 ± 8.59^A^
Epoxy archwire	67.26 ± 8.41^A^	55.27 ± 15.72^A^	59.37 ± 11.17^A^
Rhodium archwire	45.90 ± 7.51^A^	40.90 ± 10.37^A^	43.40 ± 9.51^A^

Different superscript letters by line represent statistically significant difference.

There was no statistically significant difference between the sexes regarding the aesthetic perceptions of the tested archwires (Table 3). 

**Table 3:** Descriptive statistics (mean and standard deviation) and comparison between gender (Mann-Whitney test)

	Male (n = 45) (Mean ± S.D.)	Female (n = 45) (Mean ± S.D.)	p
Steel archwire	36.33 ± 8.97	33.50 ± 10.06	0.08
NiTi archwire	30.84 ± 7.65	32.48 ± 9.02	0.44
Epoxy archwire	64.92 ± 9.86	61.55 ± 13.94	0.72
Rhodium archwire	44.71 ± 7.26	45.03 ± 11.25	0.44

## DISCUSSION

The aesthetic innovations of orthodontic appliances and the enhancement of interdisciplinary treatments options have contributed to the number of adult patients visiting orthodontic dental offices.^14^ Nowadays, orthodontists frequently consider the aesthetic implications of adult patients’ appliances. Studies have been conducted related to the mechanical and physical properties of orthodontic coated and uncoated archwires.^6^ ^,^ ^15^ ^-^ ^19^ Nevertheless, there is a scarcity of information related to aesthetic judgments about these wires when aesthetic brackets are used. No previous study has evaluated this proposal; therefore, the present results are unprecedented. The appearance of orthodontic appliance still plays an important role in patients’ decision to undergo orthodontic treatment, justifying the importance of this study. 

Laypersons were selected to evaluate the coated and uncoated archwires because they are involved with social interactions with orthodontic patients. Moreover, they are the primary consumers of dental services, as opposed to practitioners, who are the providers of care.^20^

Although orthodontic planning should be based on the esthetic demands of the patients, in the present study, the orthodontic patients were not included to avoid bias. The orthodontist could have informed them about the limitations of coated archwires regarding color changes^4^ and coating instability.^5,6^ This information could make them more analytical with regard to coated archwires, and it was speculated that it could influence their judgments, since perception has a psychological basis and is not only allied with sensation.^21^ The contact with an orthodontist and his/her opinion is a form of bias.

The present results showed that laypersons considered the metal archwires coated with epoxy resin to be desirable, complementing the aesthetic brackets of fixed orthodontic therapy.^2^ Appearance of the archwires affected the aesthetic perception when ceramic brackets are used. Previous studies have demonstrated that more aesthetic fixed orthodontic appliances are more acceptable to adult patients.^3^ ^,^ ^7^ This finding was also observed regarding the orthodontic archwires in the present study. Attractiveness appears to be related to the quantity of apparent metal in the orthodontic appliance.^2^

Rhodium is a silver-white metal that is considered an excellent light reflector. However, individuals preferred the archwires coated with epoxy resin to those coated with rhodium, possibly because they do not have any metallic aspect.

Compared to the other age groups, the age group of 31-45 years was the only one that considered NiTi archwires the least aesthetic. However, it is important to highlight that a very small difference was observed (Table 2) and most likely have no clinical significance.^2^ Moreover, to the other archwires, the age ranges demonstrated similar judgment. 

For both sexes, the aesthetic judgments of the tested archwires were similar. Pithon et al^9^ evaluated esthetic perception of black spaces between maxillary central incisors, and also did not found differences in judgments between the sexes. Probably this result is related to the fact that nowadays people want a better appearance. Moreover, the mass media plays an important role in the perception of beauty in modern culture.^22^

A limitation of this study relates to the vermillion border exposure, which was not identical for all photographs. The images used in this study could not be generated by software manipulation of one basic image, since it was necessary to change the archwires and take new photographs. Great effort was made to standardize the images. With this purpose, the volunteer remained seated and positioned in a cephalostat while the photographs were taken and during the clinical procedure of changing the archwires. Also the photographic equipment was maintained at a standardize distance. Each image was then condensed, to achieve an image with measurements identical to those on the actual patient. For this, each millimeter measured on the digital image and on the printed image was equivalent to each millimeter clinically measured on the patient, using the maxillary central incisor as the reference.^2^ ^,^ ^10^ Most part of the nose, chin and cheeks were removed to reduce the number of variables in the images.^11^ Previous similar studies,^2^ ^,^ ^7^ ^,^ ^14^ also used photographs without identical smiles. As the vermilion border change was extremely small and the laypersons were instructed to evaluate the appliance aesthetic (and not the smile), the present results can be validated.

It can be emphasized that orthodontic wires must not be selected exclusively based on aesthetic judgment. Ideally, archwires should be aesthetic and present good clinical performance.^17^ However, the coated archwires frequently undergo peeling of the superficial layer,^6^ ^,^ ^18^ so aesthetics cannot be guaranteed throughout the usual appointment interval. However, orthodontists can use aesthetic archwires in specific situations, like when the patient desires enhanced aesthetics, such as for social events, employment interviews and other similar situations. 

Studies have shown that the most aesthetically attractive materials are not those that are frequently used in orthodontic practice;^19^ current aesthetic materials must be improved, or novel ones must be fabricated with new technologies. Innovations in the aesthetic of orthodontics archwires may play an important role in acceptability of orthodontic treatment for adults.^14^

## CONCLUSIONS

The outcomes of this study demonstrated that:

» The visual aspect of ceramic brackets improved using aesthetic coating, compared to steel and NiTi archwires.
» Laypersons considered the archwires coated with epoxy resin to be the most aesthetic.
» When compared to other age groups, only the age group of 31-45 years considered the NiTi archwire to be the least aesthetic.
» The aesthetic judgments of the tested archwires were not influenced by the sex.
